# Locomotor illusions are generated by perceptual body-environment organization

**DOI:** 10.1371/journal.pone.0251562

**Published:** 2021-05-11

**Authors:** Martin Dobricki, David Weibel, Leonardo Angelini, Elena Mugellini, Fred W. Mast

**Affiliations:** 1 University of Bern, Department of Psychology, Bern, Switzerland; 2 Swiss Federal Institute for Vocational Education & Training, Learning Technologies Research Group, Zollikofen, Switzerland; 3 University of Applied Sciences of Western Switzerland, HumanTech Institute, Fribourg, Switzerland; University of Minnesota, UNITED STATES

## Abstract

While one is walking, the stimulation by one’s body forms a structure with the stimulation by the environment. This locomotor array of stimulation corresponds to the human-environment relation that one’s body forms with the environment it is moving through. Thus, the perceptual experience of walking may arise from such a locomotor array of stimulation. Humans can also experience walking while they are sitting. In this case, there is no stimulation by one’s walking body. Hence, one can experience walking although a basic component of a locomotor array of stimulation is missing. This may be facilitated by perception organizing the sensory input about one’s body and environment into a perceptual structure that corresponds to a locomotor array of stimulation. We examined whether locomotor illusions are generated by this perceptual formation of a locomotor structure. We exposed sixteen seated individuals to environmental stimuli that elicited either the perceptual formation of a locomotor structure or that of a control structure. The study participants experienced distinct locomotor illusions when they were presented with environmental stimuli that elicited the perceptual formation of a locomotor structure. They did not experience distinct locomotor illusions when the stimuli instead elicited the perceptual formation of the control structure. These findings suggest that locomotor illusions are generated by the perceptual organization of sensory input about one’s body and environment into a locomotor structure. This perceptual body-environment organization elucidates why seated human individuals experience the sensation of walking without any proprioceptive or kinaesthetic stimulation.

## Introduction

While walking through an environment, one’s body and the environment simultaneously stimulate the sensory organs [1]. The stimulation by the walking body thereby forms a structure with the stimulation by the environment. This structure is a higher-order property of stimulation [2] that is based on the simultaneous orientation of a perceptual system to multiple referents [3]. It defines a global array of stimulation [4,5] that corresponds to the human-environment relation [6] that one’s body constitutes with an environment while moving through it [7]. Thus, the perceptual experience of walking may arise from such a locomotor array of stimulation.

Human individuals can also experience walking while they are sitting. In fact, there is converging evidence that this locomotor illusion is generated [8–12] in the absence of proprioceptive [13] or kinaesthetic stimulation [14]. Hence, one can experience walking although the stimulation by one’s walking body and thus a basic component of a locomotor array of stimulation is missing. This may be facilitated by the fact that perception is organizing sensory organ input [15] into coherent perceptual events [16,17]. Thereby, perception may organize the sensory input about one’s body and environment into a perceptual structure that corresponds to a locomotor array of stimulation. We therefore examined whether locomotor illusions are generated by this perceptual formation of a locomotor structure. To this end, we exposed seated individuals simultaneously to visual stimuli, such as trees passing by, and to tactile stimuli applied to the soles of their feet. We presented these environmental stimuli differently: In one condition, the stimuli elicited the perceptual formation of a locomotor structure; in another condition, the stimuli elicited the formation of a control structure. We predicted that the perceptual formation of a locomotor structure–but not that of a control structure–would give rise to the distinct locomotor illusion of walking across an environment. Moreover, we expected that this perceptual formation would not depend on the amount of stimuli used to trigger it.

## Materials and methods

### Participants

**Fig 1:**
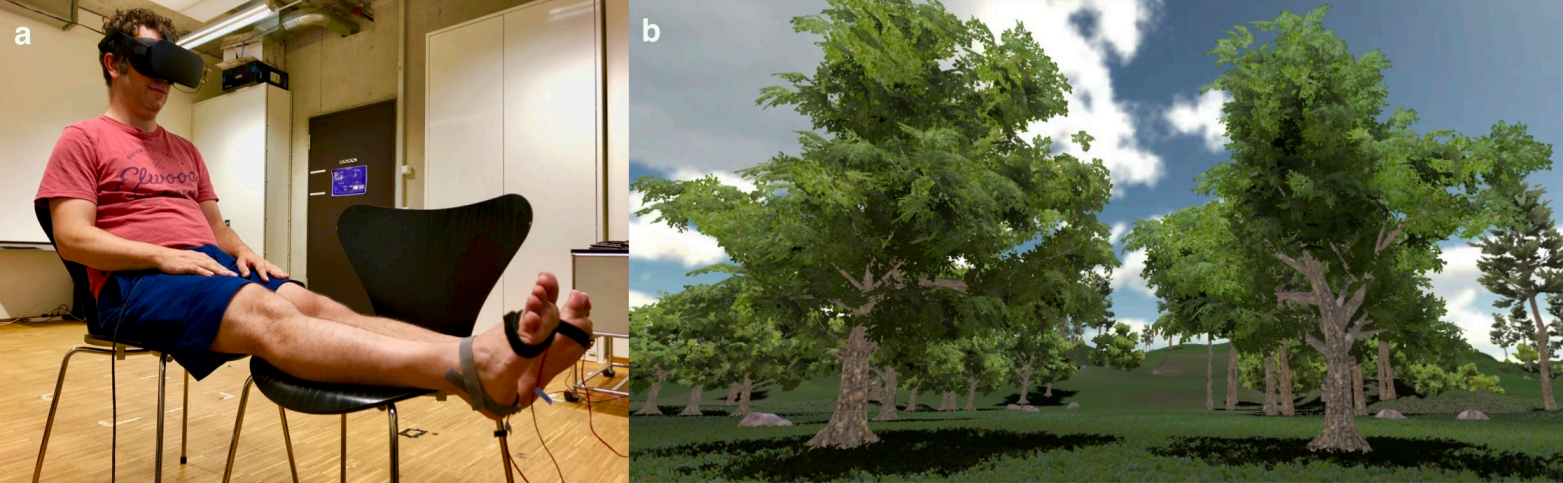
The experimental setup. (a) Seated male participant, with two vibratory devices attached to the sole of each foot, wearing a head-mounted display, via which he is being exposed to (b) a life-sized 3D virtual environment in which his virtual perspective while swaying like the head of a walking human was first moving forward across a meadow and then up a hill.

Sixteen healthy human participants (9 women, mean age = 25.1 years, SD = 2.6 years) with normal or corrected-to-normal vision participated. This sample size was chosen based on the sample size of a previous study [10] that had used an experimental treatment similar to ours. The study participants gave their written informed consent and were free to withdraw from the study at any time. None of them reported any signs of simulator sickness. The individual depicted in the photograph shown in Fig 1 provided written informed consent (by signing the PLOS consent form) for the photograph to be published in this journal. The study was approved by the Ethics Commission of the Faculty of Human Sciences of the University of Bern and was conducted in accordance with the Declaration of Helsinki.

### Stimuli and apparatus

We asked the study participants to don an Oculus Rift head-mounted display (HMD). This HMD consists of dual OLED displays with a resolution of 1200 x 1080 pixels per eye displayed at 90 Hz. It has a 94° horizontal and 93° vertical field of view. Wearing the HMD, the participants viewed a life-sized 3D virtual environment consisting of a hill and some trees (Fig 1) from a first-person, eye-level-while-walking perspective. We ran the graphics engine Unity3D on an Asus Prime Z270-K computer with an NVIDIA GeForce GTX 1070 graphics card. During the participants’ exposure to the virtual environment, the virtual perspective was simulating the head sway of a walking human [18–20] moving across a meadow for two minutes and then up to the top of a hill for another two minutes. The perspective moved either 288 m at a normal walking speed of 1.2 m per sec or 432 m at a fast walking speed of 1.8 m per sec. We programmed the virtual perspective sway to occur within a period of 579 msec in the normal-speed trials and within 485 msec in the fast-speed trials. The peak-to-peak amplitude of vertical perspective sway within this “step” time averaged 27.6 mm (SD = 3.6 mm) in the normal-speed trials and 36.0 mm (SD = 4.0 mm) in the fast-speed trials. The horizontal perspective sway amplitude was 8.5 mm on average (SD = 7.4 mm) in all trials. While exposing the participants to the motion of the swaying virtual perspective, we presented tactile stimulation to the participants using four LilyPad vibe boards, two of which were attached to the sole of each foot (Fig 1). The vibe boards were controlled by an Arduino microcontroller and vibrated with a frequency of 200 Hz and an amplitude of 0.8 G. In one condition, all four LilyPad vibe boards vibrated constantly; in the other condition, to mimic footsteps, two LilyPad vibe boards vibrated for 80 msec on each foot in alternation, with an either 579-msec pause (in normal-speed trials) or 485-msec pause (in fast-speed trials) between vibrations when the minimum of a vertical sway of the virtual perspective was reached. White noise was presented over headphones to mask any external noise.

### Experimental design

We manipulated perceptual formation (factor 1) by means of the differential tactile stimulation described above (viz., vibrations alternating from foot to foot, mimicking movement with footsteps, vs. constant vibrations to both feet, mimicking movement without footsteps): The visuo-tactile stimuli elicited either the perceptual formation of a *locomotor structure* (i.e., a structure corresponding to a body moving through an environment with footsteps) or the perceptual formation of a *control structure* (i.e., a structure corresponding to a body moving through an environment without footsteps). In addition, we manipulated the amount of stimulation (factor 2) by varying the walking speed, whereby normal walking speed resulted in a *normal* amount of stimulation and fast walking speed resulted in an *augmented* amount of stimulation. Here, the visuo-tactile stimuli were eliciting the sensory input that resulted from moving at a normal or fast walking speed. All participants received all four possible experimental combinations of perceptual formation (locomotor, control) and amount of stimulation (normal, augmented) in a 2 x 2 balanced Latin square, within-subjects design.

### Procedure

First, we asked participants to stand upright and don the HMD so that we could determine their eye level. Second, we asked participants to sit on a chair and rest their calves on another chair to relax their legs (Fig 1A). Third, the experimenter attached the vibe boards to the soles of each of the participants’ feet. The participants were then exposed to each of the four experimental trials described above for four minutes each. At the end of each trial, the participants were asked to take off the HMD and to rate their subjective experience (see next section). After providing these ratings, participants took a break for approximately two minutes.

### Psychometric ratings

The participants assessed their subjective experience by rating a set of ten self-report statements. These statements were presented in random order on a computer screen using an internet platform (www.soscisurvey.de). A visual analogue scale (VAS; min = 0; max = 100) was presented to the right of each statement. The VAS was a continuous horizontal line of about 4 cm length with the left pole labelled “not at all” and the right pole labelled “very much.” The participants were to use a computer mouse to move a small vertical line on the VAS to rate the intensity of the experience described in each statement. Participants indicated their basic sensation of moving through space by rating the following statement: “I felt like I was moving through space.” Table 1 shows the three statements concerning gait sensations used to measure locomotor illusions (Cronbach’s α = .93). It also shows the three statements used to measure other locomotion sensations (Cronbach’s α = .66). The statements “I felt like I was walking” and “I felt like I was sliding along the floor” were adapted from previous studies [8,9,11]. The other statements on gait and other locomotion sensations were newly formulated for the purpose of this study. In addition, to assess participants’ “place illusion” (i.e., illusion that they were there in the virtual environment) [21], participants rated three statements, also shown in Table 1, concerning spatial presence sensations (Cronbach’s α = .98) adapted from the MEC Spatial Presence questionnaire [22].

**Table 1:** Self-report statements used for the assessment of gait sensations, other locomotion sensations, and spatial presence sensations.

Sensation scale	Self-report statements
*Gait sensations*	I had the impression that I was placing one foot in front of the other.
	I felt like I was walking.
	I felt like my legs were moving.
*Other locomotion sensations*	I felt like I was sliding along the floor.
	I had the impression that I was riding a bicycle.
	I felt like I was being pushed in a wheelchair.
*Spatial presence sensations*	I felt like I was actually there in the virtual environment.
	It was as though my true location had shifted into the virtual environment.
	It seemed as if I was present in the virtual environment.

### Data analysis

First, the scale scores for gait sensations and spatial presence sensations were calculated for each participant in each of the four experimental conditions. This was accomplished by calculating each participant’s mean rating of the three statements used to assess the intensity of these sensations. The scale scores for gait sensations and for spatial presence sensations as well as the participants’ ratings of their basic sensation of moving through space were compared across the four experimental conditions by performing three separate two-way repeated-measures analyses of variance (ANOVAs) and by calculating the effect size $\eta_{p}^{2}$. Prior to this, we used Kolmogorov–Smirnov tests to check whether the data satisfied the normality assumption. The scale scores for gait sensations and for spatial presence sensations satisfied the normality assumption in all four experimental conditions. The ratings of the basic sensation of moving through space satisfied the normality assumption in three of four conditions. We accepted this because non-parametric analyses of these ratings did not yield different results. Second, the scale scores for gait sensations and those for other locomotion sensations were calculated for each participant in the locomotor structure trials as well as in the control structure trials. These scale scores were then used to compare the ratings of gait sensations with the ratings of other locomotion sensations in the locomotor structure trials and, separately, in the control structure trials. Due to the distribution of scores for other locomotion sensations, these comparisons were accomplished with the Wilcoxon signed-rank test (two-tailed) and by calculating the effect size *r*_contrast_ [23]. Finally, we examined whether, in the locomotor structure trials or in the control structure trials, the scale scores for gait sensations and for other locomotion sensations were correlated (Spearman’s rho, two-tailed). As for descriptive statistics, we calculated the median (*Md*) and interquartile range [IQR] for all ratings. The statistical analyses were performed with Microsoft Excel and the statistical software SPSS. The visualization of the statistical results was generated with the ggplot2 package within the statistical software R. It consisted in box-and-whisker plots as well as depicting the sixteen participants’ individual overall ratings as circles.

## Results

The score for gait sensations was about medium, indicating that participants’ gait sensations were about medium in intensity, in the locomotor structure trials, *Md* = 45.1, IQR [31.9, 59.1]. It was significantly higher in the locomotor structure trials, *F (1, 15) = 27.51, p = .000, $\eta_{p}^{2}$ = .647*, than in the control structure trials, *Md* = 13.7, IQR [6.5, 32.1]. The amount of stimulation did not have an effect, *F (1, 15) = 1.92, p = .186, $\eta_{p}^{2}$ = .114,* nor did it interact with the perceptual formation regarding the score for gait sensations, *F (1, 15) = 0.01, p = .913, $\eta_{p}^{2}$ = .001*.

The participants’ ratings of their basic sensation of moving through space were roughly equal, *F (1, 15) = 1.92, p = .186, $\eta_{p}^{2}$ = .114*, in the locomotor structure trials, *Md* = 60.3, IQR [47.8, 79.1] and the control structure trials, *Md* = 55.5, IQR [41.3, 70.0]. The amount of stimulation did not have an effect, *F (1, 15) = 0.10, p = .747, $\eta_{p}^{2}$ = .007,* nor did it interact with the perceptual formation regarding the basic sensation of moving through space, *F (1, 15) = 0.06, p = .797, $\eta_{p}^{2}$ = .005*. The score for spatial presence sensations was almost significantly, *F (1, 15) = 3.31, p = .089, $\eta_{p}^{2}$ = .181*, higher in the locomotor structure trials, *Md* = 58.8; IQR [36.9, 74.5], than in the control structure trials, *Md* = 46.5; IQR [30.8, 67.2]. The amount of stimulation did not have an effect, *F (1, 15) = 0.54, p = .473, $\eta_{p}^{2}$ = .035,* nor did it interact with the perceptual formation regarding the score for spatial presence sensations, *F (1, 15) = 0.01, p = .920, $\eta_{p}^{2}$ = .001*.

**Fig 2:**
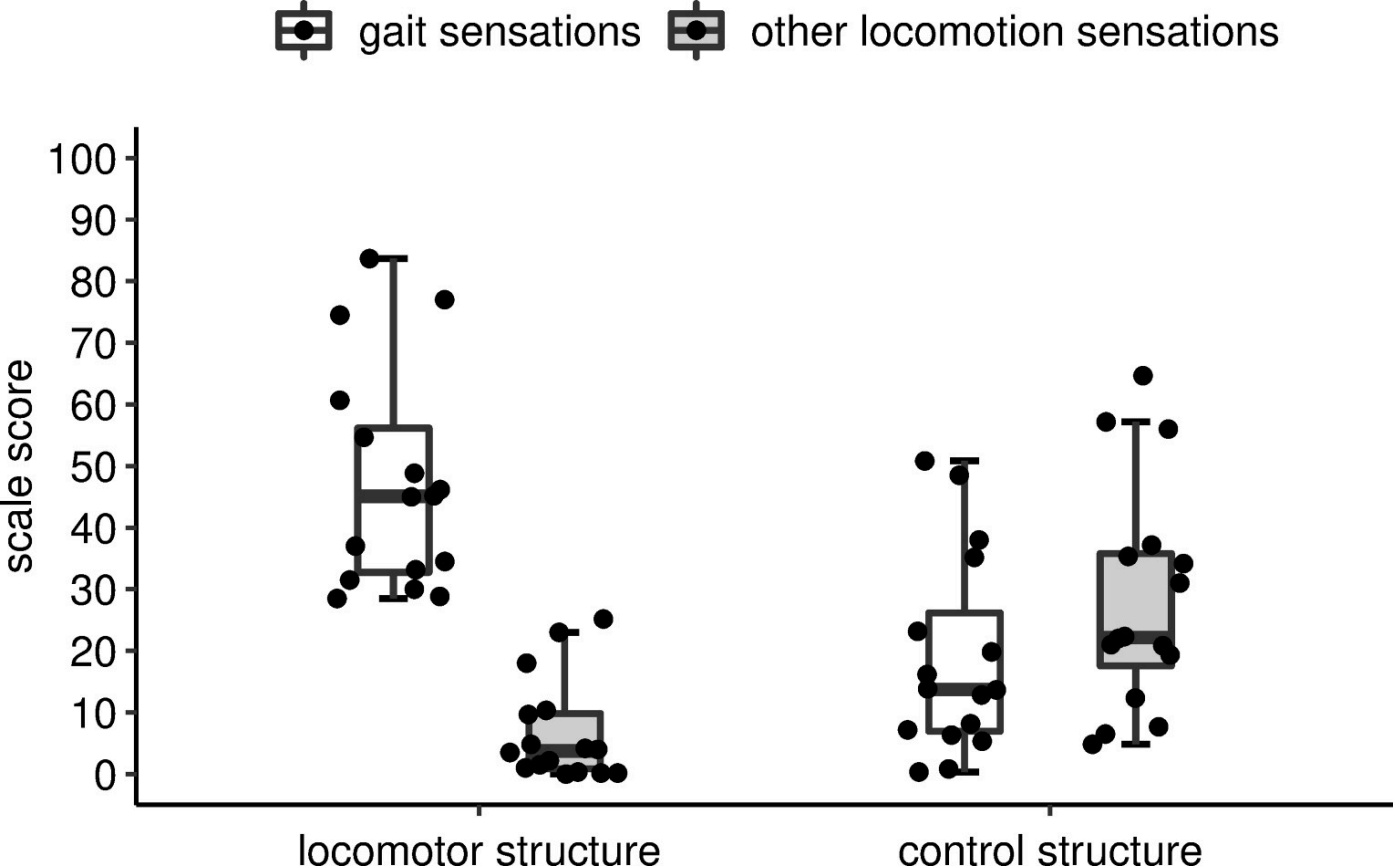
Comparison of gait sensations and other locomotion sensations. Box-and-whisker plots of the scores for gait sensations and for other locomotion sensations in the locomotor structure trials and in the control structure trials. Bold horizontal lines indicate median overall rating score; boxes indicate the lower and upper quartiles; whiskers indicate the farthest data points within 1.5 times of the lower and upper quartiles, respectively. The circles depict the individual scale scores of the 16 participants.

As depicted in Fig 2, the score for gait sensations was significantly higher than the score for other locomotion sensations in the locomotor structure trials, *Z* = -3.51, *p* = .000, *r*_contrast_ = .87, but not in the control structure trials. The score for other locomotion sensations was significantly higher in the control structure trials than in the locomotor structure trials, *Z* = -2.95, *p* = .003, *r*_contrast_ = .74 (see Fig 2). The score for gait sensations and the score for other locomotion sensations were not correlated in the locomotor structure trials, *r*_*s*_ = -.22, *p* = .402, *N* = 16, or in the control structure trials, *r*_*s*_ = -.12, *p* = .633, *N* = 16.

## Discussion

We found that our seated participants experienced more gait sensations than other locomotion sensations when they were exposed to environmental stimuli that elicited the perceptual formation of a locomotor structure. They did not experience such distinct locomotor illusions when the stimuli instead elicited the perceptual formation of the control structure. Neither finding depended on the amount of stimulation. These findings suggest that locomotor illusions are generated by the perceptual organization of sensory input about one’s body and environment into a locomotor structure. This perceptual body-environment organization elucidates why seated human individuals can experience the sensation of walking without any proprioceptive or kinaesthetic stimulation.

Walking results in concomitant sensory input such as visual, tactile or auditory input [24] about one’s body and environment. Gait sensations are understood to arise from such multisensory input. However, current theoretical perspectives on this perceptual process differ. On the one hand, gait sensations are understood to arise due to perception organizing multisensory input [15] about one’s body and environment into coherent perceptual events [16,17]. On the other hand, gait sensations are understood to arise due to perception detecting the structure of this multisensory input [3] as corresponding to a global array of stimulation [4,5]. Our findings are in line with both of these theoretical perspectives on perception in that they suggest that these perspectives concern different aspects of the same perceptual process: Gait sensations arise from the organization of multisensory input about one’s body and environment into a perceptual structure that corresponds to a global array of stimulation. This perceptual body-environment organization may also be the reason why locomotor behaviour was found to modulate both the perception of one’s walking body relative to space [25] as well as the perception of space relative to one’s walking body [26].

Walking is a motor behaviour involving the control of body movements. The sensation of such motor control [27] is understood to arise due to the brain matching actual sensory input [28] to the input that its locomotor representation predicts [29]. The actual sensory input used for this perceptual-motor integration and the sensory input giving rise to gait sensations should be the same. Hence, the perceptual body-environment organization that we found to give rise to locomotor illusions may play a key role in the emergence of the sensation of natural and artificial locomotor control [9,27] during perceptual-motor integration.

Spinal cord injury patients attempting to move their paralyzed feet were found to use the same brain network that healthy participants do when they are moving their feet [30]. We found that gait sensations arise from the perceptual formation of a locomotor structure. It is essential for the brain to form perceptual-motor structures in order to control motor action [31]. Hence, the brain may be capable of learning to combine its perceptual-motor representation of locomotor movements with sensory stimuli that elicit the perceptual formation of a locomotor structure. Accordingly, exposing spinal cord injury patients to such sensory stimuli while they are attempting to walk may be conducive to a reorganization of the neural pathways used for locomotor behaviour [32]. This procedure may bear the potential to enhance invasive treatments of individuals with incomplete spinal cord injury [33]. It may even serve as a non-invasive alternative to invasive treatments [34] providing support to patients relearning to walk.

The procedure we used to assess locomotor illusions in seated individuals was based on the psychometric measurement of this experience. It did not involve biometric measures such as those of muscular or brain activity. This might be regarded as a limitation of our study. However, it is important to consider that parameters such as muscular activity can only serve as a biometric measure of locomotor illusions if a pattern of such parameters that is specific for locomotor illusions has already been determined. For this purpose, one would have to examine whether individuals showing such a pattern are in fact experiencing locomotor illusions. Thus, it would be an invalid argument that biometric measures are required to unambiguously establish that seated individuals are experiencing locomotor illusions. In a first step, which is what our study represents, this can only be accomplished by measures like ours, that is, by psychometric measures of conscious experience.

Demand characteristics of the stimuli [35] used to induce locomotor illusions could have confounded the scores for gait sensations and for other locomotion sensations. However, these two scores would have been interrelated if this were the case, as they would both refer to a presumed demand. Hence, our finding that the participants’ scores for gait and for other locomotion sensations were not correlated may suggest that they were not confounded by demand characteristics. Nevertheless, an important avenue of future research will be to examine in more detail whether and how locomotor illusions are susceptible to demand characteristics like other bodily illusions are [36].

Finally, our findings partly indicate that locomotor illusions are accompanied by an intensified illusion of being inside the virtual environment in which the illusions are occurring. This place illusion [21] is also intensified in individuals who are moving through a virtual environment by means of physical locomotor behaviour [25]. This intensification may occur due to the perceptual organization of sensory input about one’s physical body movements and about the virtual environment to a unique locomotor structure. In this regard, human locomotor sensation and place sensation may rely on the same perceptual body-environment organization.
